# Seasonal Fluctuations and Stability of Adenosine in Dried Blood Spots for Neonatal Screening

**DOI:** 10.3390/ijns11030063

**Published:** 2025-08-13

**Authors:** Xiangchun Yang, Jing Liu, Xia Li, Dongyang Hong, Shanshan Wu, Changshui Chen, Haibo Li

**Affiliations:** 1 The Central Laboratory for Birth Defects Prevention and Control, The Affiliated Women and Children’s Hospital of Ningbo University, Ningbo 315000, China; feyangxiangchun@nbu.edu.cn; 2 Ningbo Key Laboratory for the Prevention and Treatment of Embryogenic Diseases, The Affiliated Women and Children’s Hospital of Ningbo University, Ningbo 315000, China; 3 Ningbo Key Laboratory of Genomic Medicine and Birth Defects Prevention, The Affiliated Women and Children’s Hospital of Ningbo University, Ningbo 315000, China; 4 Hunan Provincial Key Laboratory of Regional Hereditary Birth Defects Prevention and Control, Changsha Hospital for Maternal & Child Health Care Affiliated to Hunan Normal University, Changsha 410000, Chinaxiali38227429@sina.com (X.L.); 5 Department of Genetic Medicine Center, Women’s Hospital of Nanjing Medical University (Nanjing Women and Children’s Healthcare Hospital), Nanjing 210000, China; www-hdy@163.com; 6 Paediatric Surgery Centre, The Affiliated Women and Children’s Hospital of Ningbo University, Ningbo 315000, China; wsszhuhe@163.com

**Keywords:** neonatal screening, adenosine, adenosine deaminase deficiency, seasons, dried blood spot testing, specimen handling

## Abstract

Seasonal and environmental factors, including temperature, humidity, and storage conditions, significantly impact the stability of biochemical markers in dried blood spot (DBS) samples. This study investigates these influences specifically for adenosine (ADO) levels, a critical biomarker for neonatal screening of adenosine deaminase (ADA) deficiency. This study analyzed seasonal fluctuations in ADO concentrations across three regions in China (Ningbo, Nanjing, and Changsha) over 11 months, and evaluated ADO stability under different storage conditions (4 °C, 20 °C, and 40 °C). ADO levels demonstrated significant seasonal variability, peaking in July–August. Median concentrations increased by 111–189% in warmer months compared to winter across all sites. Storage experiments showed that ADO was most stable at 4 °C (fluctuations < 5% over 7 days), while levels at 40 °C increased by 18%. Re-adjusting the ADO reference range based on seasonal data reduced false positive rates from 2.48% to 0.15%, a 94% reduction. This study underscores the necessity of implementing seasonally dynamic reference ranges and strict cold-chain storage (4 °C) to enhance screening accuracy for ADA deficiency. The findings provide a robust foundation for optimizing neonatal screening protocols globally, especially in regions with distinct seasonal climates.

## 1. Introduction

The advent of tandem mass spectrometry (MS/MS) has revolutionized neonatal screening, significantly enhancing the early diagnosis of inherited metabolic disorders (IMDs) [1,2,3]. Neonatal MS/MS screening effectively detects various IMDs, including amino acid disorders, organic acidemias, and fatty acid oxidation defects [3,4,5]. Recently, the Revvity NeoBase2 non-derivatized MS/MS kit introduced ADO measurement, enabling the screening of adenosine deaminase (ADA) deficiency and expanding the scope of neonatal metabolic disorder screening [6,7].

ADA deficiency is a rare genetic disorder that leads to severe combined immunodeficiency (SCID), characterized by profound impairment of the immune system. It is caused by mutations in the *ADA* gene, resulting in the toxic accumulation of ADO in immune cells, primarily affecting T-cells, B-cells, and natural killer (NK) cells. This leads to a T–B–NK–SCID phenotype. Without early treatment such as enzyme replacement therapy or hematopoietic stem cell transplantation, affected infants experience life-threatening infections [8,9,10]. As one of the common causes of SCID, ADA deficiency has an estimated incidence of 1:375,000 to 1:660,000 live births in Europe [11]. According to Yan et al., the overall carrier frequency in the Chinese population was 1.05%, and the estimated incidence of deficiency of adenosine deaminase 2 was approximately 1 in 92,251 individuals [12]. In neonatal screening, the T-cell Receptor Excision Circle (TREC) method is commonly used to detect SCID [13,14]. Since ADA deficiency causes severe T-cell depletion, TREC serves as an indirect marker for SCID, including ADA deficiency. However, TREC screening has limitations in identifying late-onset ADA deficiency, as T-cell depletion may not significantly lower TREC levels in early life, potentially missing some cases. In contrast, the MS/MS kit directly measures ADO levels in dried blood spots (DBSs) [15,16]. Elevated ADO concentrations are a key indicator of ADA deficiency, providing a reliable screening method.

Seasonal and environmental factors, such as temperature, humidity, and storage conditions, can significantly impact the stability of biochemical markers in DBSs for neonatal screening [17,18,19,20]. However, the reliability and accuracy of ADO measurements in neonatal screening, especially regarding seasonal variations and DBS sample stability, remain understudied. Given the diverse environments of neonatal screening centers, understanding these factors’ impact on test results is crucial. For example, samples stored at room temperature or in high humidity may degrade chemically, leading to inaccurate results and affecting clinical decisions.

In our laboratory, a notable increase in median ADO levels during July and August led to a higher initial positive rate and more neonatal recalls. This prompted us to study the impact of seasonal temperature and humidity on ADO stability in DBS. This study aimed to investigate the monthly variations in ADO levels in neonatal screening samples collected over one year from three screening centers. Additionally, it aimed to assess the short-term stability of ADO in DBS samples under three storage conditions: 4 °C in a sealed bag in a refrigerator, 20 °C at room temperature with about 50% relative humidity, and 40 °C with 70% relative humidity in an incubator. By analyzing seasonal fluctuations and conducting a stability study, this research sought to provide insights into factors influencing the reliability of ADO measurements in neonatal screening.

## 2. Materials and Methods

### 2.1. Monthly Variations in ADO Levels in Neonatal Screening

We used neonatal screening data from the Central Laboratory of Birth Defects Prevention and Control at The Affiliated Women and Children’s Hospital of Ningbo University (Ningbo, China), and two other centers (Nanjing Women and Children’s Healthcare Hospital; Changsha Hospital for Maternal & Child Health Care). According to national guidelines and routine practice, heel prick sampling for DBS collection is typically performed between 48 h and 7 days after birth, most commonly in the hospital before discharge. Once collected, DBS samples are air-dried at room temperature, sealed in bags without desiccants, and stored at 2–8 °C until transported, either under cold-chain conditions or using insulated containers with ice packs, to the neonatal screening laboratory. Samples are delivered and received by the laboratory within 3–5 working days and are typically analyzed by MS/MS within 1–3 days after arrival.

**Figure 1:**
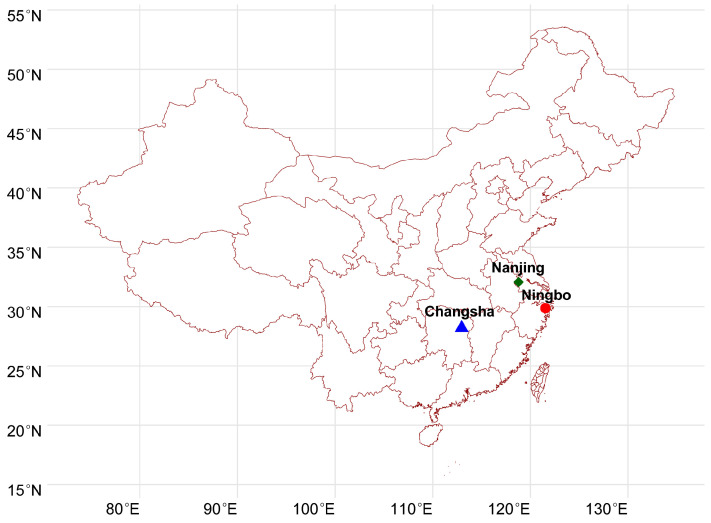
Geographic locations of the three neonatal screening centers. Ningbo, Nanjing, and Changsha are located in subtropical monsoon regions of central and eastern China.

From April 2024 to February 2025, a total of 59,339 neonatal screening specimens were analyzed at the Ningbo center, with monthly screening volumes between 4158 and 5701 specimens. During the same period, the Changsha center processed 52,905 specimens, with monthly volumes ranging from 3619 to 4952 specimens, and the Nanjing center processed 63,378 specimens, with monthly volumes between 4629 and 5823 specimens. Each center conducts over 50,000 annual screenings. Neonatal screening data generated from April 2024 to February 2025, during which the NeoBase2 non-derivatized MS/MS kit (Revvity Inc., Suzhou, China) was employed for IMD screening by MS/MS, were retrospectively analyzed. These regions in central and eastern China have a subtropical monsoon climate with distinct and similar seasonal variations (Figure 1).

### 2.2. Short-Term Stability Studies of ADO in DBSs

Venous blood from a healthy adult volunteer (a 37-year-old male of Han ethnicity) was collected to prepare DBS samples. The blood was spotted onto neonatal screening filter paper and dried at room temperature. Then, the dried blood spots were stored under three conditions to assess ADO short-term stability: 4 °C in a sealed bag without desiccants in a refrigerator, 20 °C at room temperature with about 50% relative humidity, and 40 °C with 70% relative humidity in an incubator.

ADO quantification in DBSs was performed using Xevo TQD tandem mass spectrometers (Waters Corp., Milford, MA, USA) with the NeoBase2 non-derivatized MS/MS kit, following the manufacturer’s protocol. Each analysis included blank, low-value, and high-value quality control samples. Three samples were prepared for each storage condition and analyzed in triplicate. Daily measurements were taken for seven consecutive days, generating nine data points per condition per day.

### 2.3. Statistical Analyses

Statistical analyses were conducted using SPSS Statistics 18.0 (IBM Corp., New York, NY, USA). Descriptive statistics (means, standard deviations, medians) for ADO levels were calculated. Regression analysis was used to assess the influence of environmental factors (temperature, humidity) on ADO concentrations.

### 2.4. Ethics

This study was approved by the Ethics Committee of The Affiliated Women and Children’s Hospital of Ningbo University (approval number EC2024-167, 11 December 2024). Written informed consent was obtained from the healthy volunteer for blood use in the experiment.

## 3. Results

### 3.1. Analysis of ADO Quality Control in Neonatal Screening

To eliminate the influence of experimental factors on median ADO level changes in DBS samples, we analyzed the ADO quality control (QC) data in our laboratory. We used the QC samples provided with the reagent kit following the manufacturer’s guidelines. For each 96-well plate, A1 was the extraction working solution, A2 and H11 were NeoBase2 Control Low, A3 and H12 were NeoBase2 Control High. About 48 plates were processed monthly. The monthly coefficient of variation (CV) for ADO quality control is shown in Table 1. The LC CV ranged from 6.65% to 15.0%, and the HC CV from 5.18% to 7.01%, indicating consistent testing performance. Notably, the ADO concentration in QC lot number 749559 was substantially higher than that of other lots, which may have contributed to the observed fluctuation. This confirmed that laboratory procedures were reliable and that ADO level variations were not due to detection errors.

### 3.2. Assessment of Monthly Changes in ADO Levels in Neonatal Screening

**Figure 2:**
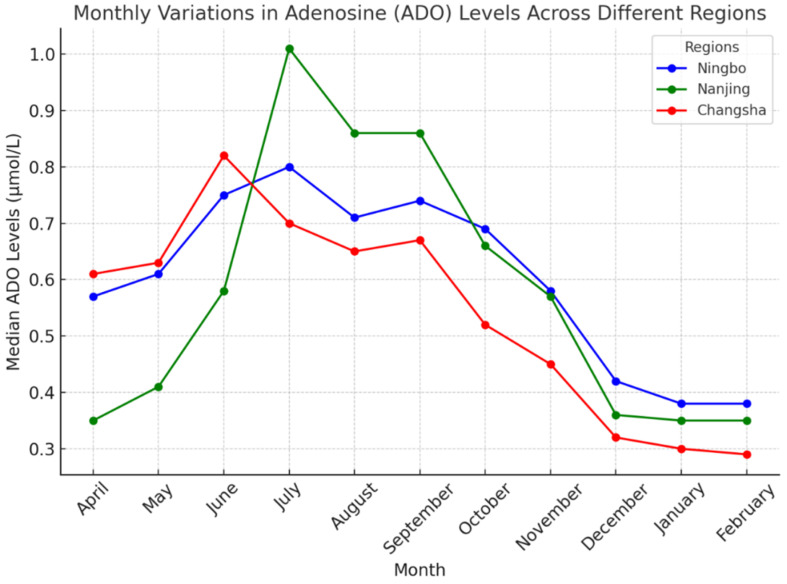
Monthly variations in ADO levels across different regions. The graph shows the median ADO concentrations (µmol/L) in neonatal DBS samples from three screening centers in Ningbo (blue), Nanjing (green), and Changsha (red) over a 11-month period, from April 2024 to February 2025. A pronounced seasonal fluctuation is observed, with ADO levels peaking in July and August, particularly in Nanjing, and showing lower levels during the colder months. The differences in seasonal patterns across regions highlight the influence of environmental factors on ADO stability in DBS samples.

We analyzed ADO concentrations in neonatal DBS samples from Ningbo, Nanjing, and Changsha between April 2024 and February 2025. As shown in Figure 2, ADO levels exhibited distinct seasonal fluctuations in each center. A significant increase in ADO concentrations during warmer months was observed across all three centers. In Ningbo, ADO levels increased from 0.38 µmol/L in January to 0.80 µmol/L in July (a 111% rise). In Nanjing, the median ADO concentration rose from 0.35 µmol/L in April to 1.01 µmol/L in July (a 189% increase). In Changsha, the lowest ADO level was 0.29 µmol/L in February, peaking at 0.82 µmol/L in June (a 183% increase).

**Figure 3:**
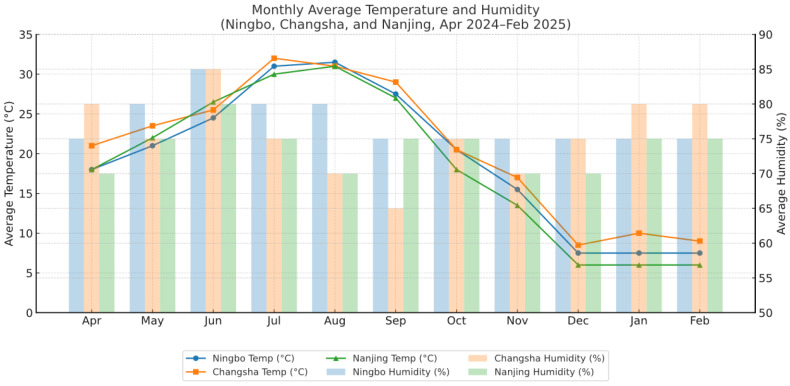
Monthly average temperature and humidity (Ningbo, Changsha, and Nanjing, April 2024–February 2025).

Detailed monthly temperature and humidity data for each center are provided in Figure 3. From April 2024 to February 2025, the average monthly relative humidity ranged from approximately 75% to 85% in Ningbo, 70% to 80% in Nanjing, and 65% to 85% in Changsha. During the same period, the average monthly temperature ranged from approximately 7.5 °C to 31.5 °C in Ningbo, 6 °C to 31 °C in Nanjing, and 8.5 °C to 32 °C in Changsha. By comparing Figure 2 and Figure 3, it is evident that the seasonal fluctuations in median ADO concentrations across Ningbo, Nanjing, and Changsha closely parallel the variations in ambient temperature.

### 3.3. Analysis of Positive Cases and Positivity Rates Under Different ADO Reference Intervals

After observing seasonal ADO concentration increases, we analyzed the impact of different ADO reference intervals on positive cases and positivity rates. Positive cases in neonatal screening, defined by ADO values exceeding the reference interval, do not confirm ADA deficiency but require further investigation.

Before officially using the NeoBase2 non-derivatized MS/MS kit, we tested about 3000 neonatal DBS samples after optimizing the assay method. The initial ADO reference interval was set between the 0.5th and 99.5th percentiles (0.10–1.14 µmol/L). After routine use until October 2024, 19,307 samples were tested, leading to a revised reference interval of 0.19–2.02 µmol/L.

As summarized in Table 2, the application of the revised ADO reference interval (0.19–2.02 µmol/L) resulted in a markedly lower number of positive cases and positivity rates compared to the original reference interval (0.10–1.14 µmol/L). In April, the positivity rate was 0.08% (2 positive cases) with the new reference interval and 0.51% (13 positive cases) with the original. This trend continued, with the new reference interval reducing positive cases and rates. In July, the positivity rate was 6.96% (337 positive cases) with the original reference interval and 0.17% (8 positive cases) with the new one. Overall, the new reference interval had 72 positive cases (0.15%) compared to 1225 (2.48%) with the original, showing that accurate reference intervals require large samples and long observations and are crucial for reliable screening.

### 3.4. Short-Term Stability of ADO in DBS with Different Storage Conditions

Based on the significant seasonal ADO level fluctuations, we investigated ADO short-term stability in DBS under different storage conditions. As shown in Table 3, ADO concentrations on Day 1 were lower at 4 °C (0.25 ± 0.03 µmol/L) than at room temperature (0.30 ± 0.04 µmol/L) and 40 °C (0.33 ± 0.05 µmol/L). Lower storage temperatures maintained lower ADO concentrations over seven days. At 4 °C, ADO levels were relatively stable, while at room temperature, they increased from 0.30 ± 0.04 µmol/L to 0.32 ± 0.04 µmol/L. At 40 °C, ADO concentrations rose more significantly, from 0.33 ± 0.05 µmol/L to 0.39 ± 0.04 µmol/L on Day 6, then decreased to 0.36 ± 0.08 µmol/L on Day 7. Paired-sample *t*-tests were performed to assess the statistical significance of ADO differences across storage conditions. ADO concentrations at 40 °C were significantly higher than those at 4 °C on multiple days (all *p* < 0.05). Significant differences between room temperature and 40 °C were observed on Days 4 and 5 (*p* < 0.05). A significant difference between 4 °C and room temperature emerged on Day 7 (*p* = 0.0084), while no significant differences were found between these two conditions on earlier days.

**Figure 4:**
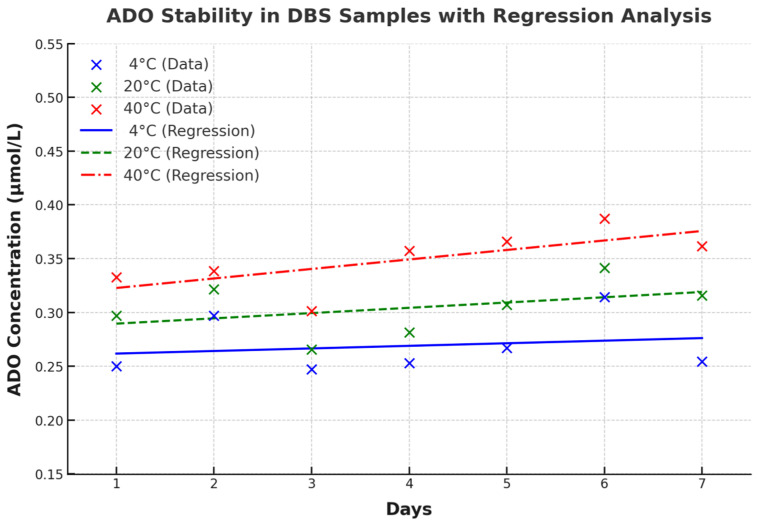
ADO stability in DBS samples with regression analysis. Regression analysis of ADO concentrations in DBS samples over a seven-day period under three storage conditions (4 °C, 20 °C, and 40 °C). Data points represent mean concentrations for each day, while the regression lines illustrate trends in ADO stability. At 4 °C, the slope was 0.0024 µmol/L/day, with an intercept of 0.2594 µmol/L, an R-squared value of 0.0387, and a *p*-value of 0.6725, indicating minimal variation in ADO levels. At 20 °C, the slope increased to 0.0049 µmol/L/day, with an intercept of 0.2847 µmol/L, an R-squared value of 0.1734, and a *p*-value of 0.3527, suggesting an observed trend towards increasing ADO levels. At 40 °C, the slope was the steepest at 0.0088 µmol/L/day, with an intercept of 0.3139 µmol/L, an R-squared value of 0.4755, and a *p*-value of 0.0865, reflecting a stronger trend in ADO concentration change over time.

As shown in Figure 4, regression analysis revealed that at 4 °C, the slope was 0.0024 μmol/L/day, indicating minimal change (R-squared = 0.0387, *p* = 0.6725). At 20 °C, the slope increased to 0.0049 μmol/L/day (R-squared = 0.1734, *p* = 0.3527), and at 40 °C, it was 0.0088 μmol/L/day (R-squared = 0.4755, *p* = 0.0865), demonstrating an observed trend toward increasing ADO levels with higher storage temperatures.

## 4. Discussion

A substantial body of research has delved into the detection of IMDs via MS/MS, with a particular emphasis on screening amino acids, acylcarnitines, and other metabolites [21,22]. These investigations have explored diverse factors, including prematurity, storage conditions, gender, and age, and their impacts on the levels of specific biomarkers in neonates [17,18,20,23]. Moreover, the effects of sample storage conditions and age-related metabolic shifts, as well-documented in the literature, influence the reliability of screening results [23]. Previous studies have demonstrated that environmental fluctuations and sample storage conditions can significantly affect the performance of newborn screening. Supriya et al. highlighted the importance of maintaining appropriate ambient temperature during DBS preparation and storage to avoid false positive results when screening for lysosomal storage disorders [24]. Similarly, Kloosterber et al. reported seasonal variations in immunoreactive trypsinogen levels in DBS, further underscoring the potential impact of environmental factors on screening outcomes [25].

However, while the variations in these factors for amino acids, acylcarnitines, and other traditional screening biomarkers have been thoroughly investigated, relatively few studies have specifically focused on the newly incorporated ADO marker, which is part of the NeoBase2 kit used for detecting metabolic disorders. There is a research gap regarding how other factors, such as seasonal variations, temperature, and humidity fluctuations, affect ADO concentrations in DBS during neonatal screening.

To address this gap, we analyzed ADO levels in three Chinese regions with distinct seasonal climates. Our study confirmed significant seasonal fluctuations in ADO levels, with higher median concentrations observed in July and August. Comparison of ADO patterns with environmental data (Figure 2 and Figure 3) shows that ADO fluctuations closely follow changes in ambient temperature. These findings suggest that seasonal temperature variation is likely the primary environmental factor influencing median ADO concentrations in DBS samples. However, it remains uncertain whether this relationship between ADO fluctuations and temperature variability holds true in regions with different climatic characteristics. For example, Kunming in southwest China, located on a low-latitude plateau, experiences minimal annual temperature variation (approximately 10 °C) and maintains a spring-like climate year-round. In contrast, Turpan in northwest China exhibits not only significant annual temperature variation but also large diurnal temperature differences (up to 20 °C between day and night). Whether ADO concentrations in such distinct climates exhibit similar or different seasonal patterns warrants further investigation. Multi-center studies across diverse climatic zones would be valuable for validating these findings and enhancing the accuracy and applicability of ADO reference intervals in neonatal screening programs.

One consequence of seasonal fluctuations in ADO levels is a potential increase in the number of screen-positive cases during warmer months. These findings further underscore the importance of establishing an appropriate reference interval for ADO. As demonstrated in the results of Table 2, an inappropriately defined reference interval may lead to an unacceptably high number of positive cases, which is inconsistent with the extremely low incidence of ADA-SCID.

One potential explanation is that warmer ambient temperatures may accelerate metabolic processes, leading to an increase in ADO production [26,27,28]. Higher temperatures could result in more rapid cellular activity, including the breakdown of ATP and the subsequent generation of ADO. It is not yet known whether these fluctuations reflect genuine physiological changes in neonatal metabolism, temperature-dependent biochemical processes, or artifacts related to sample collection, drying, storage, and transport conditions. Red blood cells (RBCs) may undergo lysis during these stages, with the extent of lysis potentially influenced by environmental conditions. Jimmerson et al. have shown that there is a difference in nucleotide triphosphate and monophosphate concentrations in DBSs compared to RBC lysates. They suggest there is hydrolysis of the triphosphate in DBSs [29]. Townsend et al. reported significant seasonal differences in purine and pyrimidine concentrations between summer and winter in adults, indicating that temperature and seasonality can influence nucleotide metabolism [30].

Additionally, it is reasonable to assume that newborns’ physiological responses to seasonal temperature changes, such as alterations in metabolic rate or enzyme activity, could contribute to changes in ADO concentrations. This necessitates further investigation into the biological mechanisms underlying these variations, including potential shifts in cellular metabolic pathways or enzymatic activities, such as those of ADA, which is known to play a crucial role in ADO metabolism.

To better understand the behavior of ADO concentrations in DBS samples under different storage conditions, we conducted a short-term stability study. Our results demonstrated that ADO concentrations remained relatively stable at 4 °C over the seven-day period, while an upward trend was observed at room temperature (~20 °C) and became more pronounced at 40 °C. Although these changes did not reach statistical significance within the short duration of the study, it is possible that extending the storage period would reveal more evident and statistically significant trends. However, by comparing ADO concentrations under different storage conditions on the same day, we found that ADO concentrations at 40 °C were significantly higher than those at 4 °C on multiple days (*p* < 0.05), with differences becoming more pronounced as storage time increased. Additionally, a statistically significant difference between 4 °C and room temperature (20 °C) emerged on Day 7. These findings suggest that storage temperature may have a greater influence on ADO concentration changes than storage duration within the short-term window studied. One important limitation of our short-term stability study is the use of venous blood from a single healthy adult, which limits the ability to assess biological variability. Future studies involving multiple donors and extended storage durations are needed to enhance the generalizability and robustness of ADO stability findings under varying environmental conditions.

Future studies should enroll cohorts across diverse climates to validate seasonal effects. Simultaneously, expanding the scale of neonatal screening for ADA-SCID would help identify additional *ADA* gene variants, including rare or previously unreported mutations. The seasonal fluctuations in ADO levels in neonatal screening samples, as previously discussed, raise a crucial question about how to account for these variations when interpreting screening results. One approach could be to modify the ADO reference range on a monthly or seasonal basis, aligning it with fluctuations in ambient temperature and humidity. Another potential strategy to mitigate the impact of seasonal and environmental fluctuations on ADO levels is the adoption of a floating cutoff, whereby specimens are deemed positive if their ADO concentrations exceed a certain percentage above the daily or periodic median. Such an adaptive approach could help account for seasonal variations, potentially reducing false positives without compromising sensitivity. Although not yet implemented in our current screening practice, future research could analyze screening data to assess potential benefits.

## 5. Conclusions

In conclusion, temperature and humidity significantly impact ADO stability in DBS samples, with warmer months leading to elevated concentrations and higher false positive rates. By adopting seasonally adjusted reference ranges and strict temperature controls, neonatal screening programs can improve ADA deficiency detection accuracy. Further research should explore the physiological basis of ADO fluctuations and expand geographical coverage to refine screening protocols globally.

## Figures and Tables

**Table 1:** Quality control analysis of ADO levels in neonatal screening.

Month	LC Average (µmol/L)	LC SD (µmol/L)	LC CV (%)	HC Average (µmol/L)	HC SD (µmol/L)	HC CV (%)	QC Lot No.
April	0.740	0.071	9.64	4.56	0.236	5.18	744302
May	0.815	0.104	12.8	5.05	0.354	7.01	746505
June	0.833	0.094	11.3	5.16	0.327	6.34	746505
July	0.783	0.092	11.8	4.83	0.337	6.97	746505
August	2.69	0.186	6.91	7.31	0.451	6.17	749559
September	2.67	0.179	6.68	7.21	0.425	5.90	749559
October	2.69	0.179	6.65	7.53	0.450	5.98	749559
November	2.75	0.187	6.81	7.48	0.402	5.38	749559
December	0.571	0.083	14.6	4.79	0.282	5.89	750938
January	0.565	0.073	12.9	4.69	0.306	6.51	750938
February	0.587	0.088	15.0	4.73	0.285	6.02	750938

LC: low control; HC: high control; CV: coefficient of variation; QC: quality control.

**Table 2:** Comparison of the number of positive cases and positivity rates under different ADO reference interval (0.19–2.02 µmol/L and 0.10–1.14 µmol/L) across different months.

ADO Reference Interval	0.19–2.02 µmol/L		0.10–1.14 µmol/L	
	Number of Positives (*n*)	Positive Rate (%)	Number of Positives (*n*)	Positive Rate (%)
April	2	0.08	13	0.51
May	19	0.4	94	1.95
June	7	0.15	197	4.21
July	8	0.17	337	6.96
August	5	0.1	169	3.3
September	10	0.2	182	3.63
October	6	0.11	132	2.33
November	2	0.04	66	1.21
December	4	0.07	17	0.3
January	5	0.11	16	0.34
February	1	0.11	2	0.21
Total	72	0.15	1225	2.48

**Table 3:** Short-term stability of ADO in dried blood spots (DBSs) under different storage conditions: 4 °C, room temperature (20 °C with ~50% humidity), and 40 °C (with 70% humidity) over a 7-day period. The data are presented as mean ± standard deviation (µmol/L) for each day of measurement.

	4 °C	20 °C	40 °C	4 °C vs. 20 °C (*p*-Value)	4 °C vs. 40 °C (*p*-Value)	20 °C vs. 40 °C (*p*-Value)
Day 1	0.25 ± 0.03	0.30 ± 0.04	0.33 ± 0.05	0.0704	0.0022 *	0.2657
Day 2	0.30 ± 0.03	0.32 ± 0.04	0.34 ± 0.04	0.1661	0.0395 *	0.2695
Day 3	0.25 ± 0.03	0.27 ± 0.03	0.30 ± 0.07	0.3119	0.1297	0.2128
Day 4	0.25 ± 0.02	0.28 ± 0.04	0.36 ± 0.08	0.0971	0.0152 *	0.0374 *
Day 5	0.27 ± 0.03	0.31 ± 0.05	0.37 ± 0.08	0.2047	0.0290 *	0.0353 *
Day 6	0.31 ± 0.04	0.34 ± 0.00	0.39 ± 0.04	0.2719	0.0149 *	0.0738
Day 7	0.25 ± 0.03	0.32 ± 0.04	0.36 ± 0.08	0.0084 *	0.0310 *	0.1908

* *p* < 0.05; statistical comparisons were performed using paired *t*-tests.

## Data Availability

The data presented in this study are available on request from the corresponding author due to ethical and privacy restrictions.

## Abbreviations

The following abbreviations are used in this manuscript:

IMDs	Inherited metabolic disorders
ADO	Adenosine
DBS	Dried blood spot
MS/MS	Tandem mass spectrometry
ADA	Adenosine deaminase
SCID	Severe combined immunodeficiency
TREC	T-cell Receptor Excision Circle
QC	Quality control
CV	Coefficient of variation

## References

[1] Tang C., Tan M., Xie T., Tang F., Liu S., Wei Q., Liu J., Huang Y. (2021). Screening for neonatal inherited metabolic disorders by tandem mass spectrometry in Guangzhou. Zhejiang Da Xue Xue Bao Yi Xue Ban.

[2] Ruoppolo M., Malvagia S., Boenzi S., Carducci C., Dionisi-Vici C., Teofoli F., Burlina A., Angeloni A., Aronica T., Bordugo A. (2022). Expanded Newborn Screening in Italy Using Tandem Mass Spectrometry: Two Years of National Experience. Int. J. Neonatal Screen..

[3] Kononets V., Zharmakhanova G., Balmagambetova S., Syrlybayeva L., Berdesheva G., Zhussupova Z., Tautanova A., Kurmambayev Y. (2025). Tandem mass spectrometry in screening for inborn errors of metabolism: Comprehensive bibliometric analysis. Front. Pediatr..

[4] Ma S., Guo Q., Zhang Z., He Z., Yue A., Song Z., Zhao Q., Wang X., Sun R. (2020). Expanded newborn screening for inborn errors of metabolism by tandem mass spectrometry in newborns from Xinxiang city in China. J. Clin. Lab. Anal..

[5] Zhang H., Wang Y., Qiu Y., Zhang C. (2022). Expanded newborn screening for inherited metabolic disorders by tandem mass spectrometry in a northern Chinese population. Front. Genet..

[6] Lee B., Heo W.Y., Kim J.A., Lee H.S., Hwang N., Park H.D., Sung S.I., Chang Y.S., Park W.S., Lee S.Y. (2023). Comprehensive Evaluation of the NeoBase 2 Non-derivatized MSMS Assay and Exploration of Analytes with Significantly Different Concentrations Between Term and Preterm Neonates. Ann. Lab. Med..

[7] Wan Z., Liu W., Zhai Y., Ma Z., Cao Z. (2024). Performance Validation of the NeoBase 2 Non-Derivatized MSMS Assay Kit and Cutoff Values Establishment of Term and Preterm Neonates. Fetal Pediatr. Pathol..

[8] Aranda C.S., Gouveia-Pereira M.P., da Silva C.J.M., Rizzo M., Ishizuka E., de Oliveira E.B., Condino-Neto A. (2024). Severe combined immunodeficiency diagnosis and genetic defects. Immunol. Rev..

[9] Flinn A.M., Gennery A.R. (2018). Adenosine deaminase deficiency: A review. Orphanet J. Rare Dis..

[10] Hershfield M., Tarrant T., Adam M.P., Feldman J., Mirzaa G.M., Pagon R.A., Wallace S.E., Amemiya A. (1993). Adenosine Deaminase Deficiency. GeneReviews^®^ [Internet].

[11] Sauer A.V., Brigida I., Carriglio N., Aiuti A. (2012). Autoimmune dysregulation and purine metabolism in adenosine deaminase deficiency. Front. Immunol..

[12] Yan L., Sun X., Lou B., Zhang Y., Zhuang D., Jia J., Zhang L., He Y., Xu L., Wu S. (2024). Carrier frequency and incidence estimation of deficiency of adenosine deaminase 2 in the Chinese population based on massive exome sequencing data. Clin. Immunol..

[13] van der Spek J., Groenwold R.H., van der Burg M., van Montfrans J.M. (2015). TREC Based Newborn Screening for Severe Combined Immunodeficiency Disease: A Systematic Review. J. Clin. Immunol..

[14] Shinwari K., Bolkov M., Tuzankina I.A., Chereshnev V.A. (2021). Newborn Screening through TREC, TREC/KREC System for Primary Immunodeficiency with limitation of TREC/KREC. Comprehensive Review. Antiinflamm Antiallergy Agents Med. Chem..

[15] Hartog N., Hershfield M., Michniacki T., Moloney S., Holsworth A., Hurden I., Fredrickson M., Kleyn M., Walkovich K., Secord E. (2022). Newborn tandem mass spectroscopy screening for adenosine deaminase deficiency. Ann. Allergy Asthma Immunol..

[16] la Marca G., Giocaliere E., Malvagia S., Funghini S., Ombrone D., Della Bona M.L., Canessa C., Lippi F., Romano F., Guerrini R. (2014). The inclusion of ADA-SCID in expanded newborn screening by tandem mass spectrometry. J. Pharm. Biomed. Anal..

[17] Hu L., Hu Z., Yang J., Zhang Y., Shi Y., Zhu S., Yang R., Huang X. (2020). Effects of delivery and storage conditions on concentrations of amino acids and carnitines in neonatal dried blood spots. Zhejiang Da Xue Xue Bao Yi Xue Ban.

[18] Shimada Y., Kawano N., Goto M., Watanabe H., Ihara K. (2022). Stability of amino acids, free and acyl-carnitine in stored dried blood spots. Pediatr. Int..

[19] Dijkstra A.M., de Blaauw P., van Rijt W.J., Renting H., Maatman R., van Spronsen F.J., Maase R.E., Schielen P., Derks T.G.J., Heiner-Fokkema M.R. (2023). Important Lessons on Long-Term Stability of Amino Acids in Stored Dried Blood Spots. Int. J. Neonatal Screen..

[20] van Rijt W.J., Schielen P., Özer Y., Bijsterveld K., van der Sluijs F.H., Derks T.G.J., Heiner-Fokkema M.R. (2020). Instability of Acylcarnitines in Stored Dried Blood Spots: The Impact on Retrospective Analysis of Biomarkers for Inborn Errors of Metabolism. Int. J. Neonatal Screen..

[21] Millington D.S. (2024). How mass spectrometry revolutionized newborn screening. J. Mass. Spectrom. Adv. Clin. Lab..

[22] Mak C.M., Lee H.C., Chan A.Y., Lam C.W. (2013). Inborn errors of metabolism and expanded newborn screening: Review and update. Crit. Rev. Clin. Lab. Sci..

[23] He F., Yang R., Huang X., Tian Y., Pei X., Bohn M.K., Zou L., Wang Y., Li H., Wang T. (2021). Reference Standards for Newborn Screening of Metabolic Disorders by Tandem Mass Spectrometry: A Nationwide Study on Millions of Chinese Neonatal Populations. Front. Mol. Biosci..

[24] Supriya M., De T., Christopher R. (2018). Effect of temperature on lysosomal enzyme activity during preparation and storage of dried blood spots. J. Clin. Lab. Anal..

[25] Kloosterboer M., Hoffman G., Rock M., Gershan W., Laxova A., Li Z., Farrell P.M. (2009). Clarification of laboratory and clinical variables that influence cystic fibrosis newborn screening with initial analysis of immunoreactive trypsinogen. Pediatrics.

[26] Fredholm B.B., Johansson S., Wang Y.Q. (2011). Adenosine and the regulation of metabolism and body temperature. Adv. Pharmacol..

[27] Porkka-Heiskanen T., Kalinchuk A.V. (2011). Adenosine, energy metabolism and sleep homeostasis. Sleep. Med. Rev..

[28] Lowy B.A., Williams M.K. (1966). Studies on the metabolism of adenosine and adenine in stored and fresh human erythrocytes. Blood.

[29] Jimmerson L.C., Bushman L.R., Ray M.L., Anderson P.L., Kiser J.J. (2017). A LC-MS/MS Method for Quantifying Adenosine, Guanosine and Inosine Nucleotides in Human Cells. Pharm. Res..

[30] Townsend M.K., Bao Y., Poole E.M., Bertrand K.A., Kraft P., Wolpin B.M., Clish C.B., Tworoger S.S. (2016). Impact of Pre-analytic Blood Sample Collection Factors on Metabolomics. Cancer Epidemiol. Biomark. Prev..

